# Medication Adherence among Patients with Non-communicable Diseases in a Tertiary Hospital: A Descriptive Cross-sectional Study

**DOI:** 10.31729/jnma.8650

**Published:** 2024-07-31

**Authors:** Bharati Sharma, Sabita Karki, Jyoti Bhetwal, Akriti Shree Dahal

**Affiliations:** 1 Bir Hospital Nursing Campus, National Academy of Medical Sciences, Mahaboudha, Kathmandu, Nepal; 2 Bir Hospital, National Academy of Medical Sciences, Mahaboudha, Kathmandu, Nepal; 3 Ministry of Health, Koshi Province, Nepal

**Keywords:** *medication*, *non-adherence*, *non-communicable disease*

## Abstract

**Introduction:**

Non-communicable diseases (NCDs) are a leading cause of mortality, with a projected rise from 38 million in 2012 to 52 million by 2030. Among NCDs, hypertension, diabetes and Chronic Obstructive Pulmonary Disease are the major burdens in healthcare today, requiring long-term therapies and a significant effort in maintaining treatment adherence.

**Methods:**

A descriptive cross-sectional study design was adopted to determine medication adherence among patients with non-communicable diseases using non-probability, consecutive sampling techniques after ethical approval from same institute (Reference number: 524). Medication adherence was assessed on 322 patients attending the outpatient department, using a structured interview schedule, after getting Ethical approval from the Institution Review Committee. Morisky medication adherence scale, Culig adherence Scale, and Beliefs about Medications (BMQ) tool were used to determine the adherence level, causes of non-adherence and belief in medication respectively. Data was coded and analysed using SPSS version 16. Descriptive statistics were used to summarise the data.

**Results:**

The study population exhibited a mean age of 58 ± 12.80 years, with male participants 190 (59.01%). The present study revealed that 148 (45.96%) of the participants have a high adherence level to prescribed medication, and 246 (76.40%) strongly believed that without medication they would be very sick and life would be impossible.

**Conclusions:**

The study found that less than half of participants fully adhered to prescribed medicine, with forgetfulness identified as a primary cause of non-compliance.

## INTRODUCTION

Non-communicable diseases (NCDs) represent a significant global health challenge, with mortality rates projected to escalate from 38 million in 2012 to a troubling 52 million by 2030.^1^ Among the spectrum of NCDs, cardiovascular diseases, cancer, chronic respiratory disorders, and diabetes collectively contribute to a staggering 80% of NCDs related fatalities.^2^

Primary prevention activities, early detection and provision of essential care interventions for NCDs play a pivotal role in alleviating the NCDs' burden. Nepal has also substantially invested in the package of essential NCDs in an attempt to implement essential NCD interventions.^3,4^ However, a persistent challenge prevails - poor adherence to prescribed treatments even after the diagnosis of NCDs and initiation of drug therapy.^5,6^

Exploring the status of adherence to medications is a fundamental step for preventing complications related to poor adherence.^7^ Thus, this study aims to determine medication adherence among patients with non-communicable diseases.

## METHODS

A descriptive cross-sectional study design was employed to determine medication adherence among patients with non-communicable diseases. The study was conducted in the outpatient department of the National Academy of Medical Sciences (NAMS), Bir Hospital, a tertiary-level government hospital in Kathmandu. Ethical approval was received from the Institutional Review Committee of NAMS with (Reference number 524) on 26th February 2021 and data collection was started from 28 March and carried upto 21 April,2021. Informed consent was obtained from each participant after explaining the objective of the study before data collection.

The study population comprised patients diagnosed with hypertension, type II diabetes, and Chronic Obstructive Pulmonary Disease (COPD), who had been prescribed medication for more than 6 months. The sample size of the study was estimated based on the prevalence method at a 95% confidence limit and 5% allowable error; the prevalence of non-adherence was 29.8%.^8^

The sample size was calculated using the formula:


$$\begin{array}{ll} \text{n} & ={\text{Z}}^{2}\times \frac{\text{p}\times \text{q}}{{\text{e}}^{\text{2}}}\\ & =1.96^{2}\times \frac{0.298\times 0.702}{0.05^{2}}\\ & =322 \end{array}$$

Where,

n = minimum required sample size
Z = 1.96 at 95% Confidence interval (CI)
p = prevalence of altered passive eruption (29.8%) taken from a previous study^8^
q = 1-p
e = margin of error, 5%

Non-probability consecutive sampling technique was used to select the sample. A structured interview schedule was used for data collection, which consists of "Morisky Medication Adherence Scale (MMAS-4)" to assess medication adherence, "Adherence Scale Culig questionnaire" to determine causes of non-adherence, and "Beliefs about Medications (BMQ) questionnaire" to evaluate medication beliefs.^9-11^ To ensure cultural and linguistic appropriateness, all these tools were translated from English to Nepali and then back-translated to English, preserving the original meaning of the questions.

Data were collected through face-to-face interviews in a separate room within the outpatient departments (i.e., cardiac, endocrine, and respiratory OPD). To maintain confidentiality, all personally identifiable information (PII) was encrypted; a coding system was employed, assigning unique codes to each participant instead of names, ensuring participant privacy. Data completeness and consistency were reviewed, before entering it into a Google Sheet for storage. The data was then transferred to Statistical Package for Social Science (SPSS) version 16 for analysis. Descriptive statistics, including frequency, percentage, mean, and standard deviation, were used to summarise the findings

## RESULTS

Out of the 322 participants, 166 (50.72%) were in 41-60 age group, and 190 (59.01%) were male (Table 1).

**Table 1:** Socio-demographic Characteristics of the Participants (n= 322).

Demographic characteristics	n (%)
**Age (in completed years)**	
20-40	32 (9.94)
41-60	166 (51.56)
61-80	114 (35.40)
81 and above	10 (3.10)
**Sex**	
Male	190 (59.01)
Female	132 (40.99)
**Religion**	
Hindu	214 (66.46)
Buddhist	51 (15.84)
Kirat	10 (3.11)
Muslim	22 (6.83)
Christian	20 (6.21)
Others	5 (1.55)
**Ethnicity**	
Brahmin/Chhetri	147 (45.65)
Aadibasi/Janjati	115 (35.71)
Dalit	16 (4.97)
Muslim	22 (6.83)
Madhesi	10 (3.11)
Others	12 (3.73)
**Types of Family**	
Single	177 (54.97)
Joint	145 (45.03)
**Marital Status**	
Never married	8 (2.49)
Married	258 (80.12)
Divorced	6 (1.86)
Widow	50 (15.53)
**Education**	
Illiterate	131 (40.68)
Literate	191 (59.32)
**Among Literate (N=191)**	
Informal	37 (19.37)
Primary	28 (14.66)
Secondary	43 (22.52)
Higher secondary	46 (24.08)
Bachelor and above	37 (19.37)

**Figure 1:**
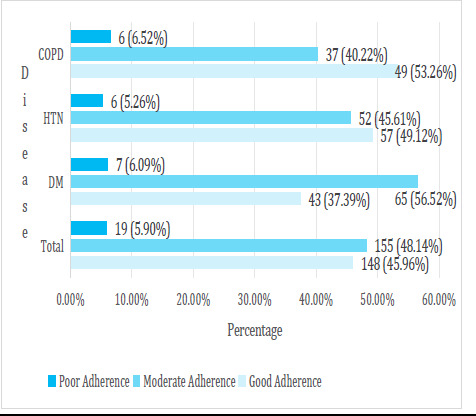
Self-reported medication adherence behaviour of participants as determined by the Morisky Medication Adherence Scale (MMAS-4).

The level of medication adherence behaviour of 322 participants as measured by the Morisky Medication Adherence Scale (MMAS-4) illustrates that 148 (45.96%) of participants reported high adherence, 155 (48.14%) reported medium adherence, and 19 (5.90%) reported low adherence to their prescribed medications (Figure 1).

The top five reasons for medication nonadherence by ranking were: 'Just forgot' 149 (49.38%), 'Not at home' 96 (29.81%), 'Consumed all medicines' 76 (23.6%), 'Shortage of supply' 67 (20.81%) and 'Sleepy at the time of medication' 65 (20.19%). Amongst those who reported, 'forgetting to take medicine' 71 (61.74%) were diabetes, 60 (52.17%) were hypertensive and 28 (30.43%) were COPD patients. (Table 2)

**Table 2:** Reason for medication non-Adherence in the study participants (n=322).

Reason for skipping drug doses	Total participants (N=322)	Participants with diabetes mellitus (N=115)	Participants with hypertension (N=115)	Participants with COPD (N=92)
	**n (%)**	**n (%)**	**n (%)**	**n (%)**
Just Forgot	159 (49.38)	71 (61.74)	60 (52.17)	28 (30.43)
Not at home	96 (29.81)	35 (30.43)	44 (38.26)	17 (18.48)
Consumed all medicines	76 (23.6)	31 (26.96)	31 (26.96)	14 (15.22)
Problem with time	33 (10.25)	12 (10.43)	17 (14.78)	4 (4.35)
Multiple drugs in a day	38 (11.8)	15 (13.04)	12 (10.43)	11 (11.96)
Shortage of supply	67 (20.81)	24 (20.87)	31 (26.96)	12 (13.04)
Feeling well	49 (15.22)	24 (20.87)	15 (13.04)	10 (10.87)
Avoid side effects	46 (14.29)	24 (20.87)	11 (9.57)	11 (11.96)
Frequently changed my therapy	31 (9.63)	14 (12.17)	15 (13.04)	2 (2.17)
Toxic drug feelings	8 (2.48)	1 (0.87)	2 (1.74)	5 (5.43)
Sleepy at the time of medication	65 (20.19)	23 (20)	16 (13.91)	26 (28.26)
Fear of Drug dependency	18 (5.59)	6 (5.22)	7 (6.09)	5 (5.43)
Expensive drug	17 (5.28)	10 (8.7)	2 (1.74)	5 (5.43)
Didn't want other people to see	14 (4.35)	4 (3.48)	3(2.61)	7 (7.61)

Didn't want other people to see14 (4.35)4 (3.48)3(2.61)7 (7.61)Regarding the participants' responses to statements related to their perception of medication necessity and concerns, 302 (93.79%) of the total participants stated that their current health depended on their medicines, and felt that their medicines protected them from deteriorating 314 (97.52%). In contrast, 136 (42.24%) of the total participants expressed concerns about having to take medicines, and around 141 (43.79%) reported worries about the long-term effects of their medications. The concern about becoming too dependent on medicines was reported by 136 (42.24%) of the total participants, and unpleasant side effects from medications were noted by 135 (41.93%) (Table 3).

**Table 3:** Participants agreeing/strongly agreeing with the "Beliefs about Medications (BMQ) Tool" statements.

Statements	Total Participants (N=322)	Participants with Diabetes mellitus (N=115)	Participants with Hypertension (N=115)	Participants with COPD (N=92)
	**n (%)**	**n (%)**	**n (%)**	**n (%)**
**Necessity Scale**				
My health at the present depends on my medicines	302 (93.79)	107 (93.04)	105 (91.3)	90 (97.83)
My life would be impossible without my medicines	246 (76.4)	82 (71.3)	86 (74.78)	78 (84.78)
Without my medications I would be very sick	263 (81.68)	88 (76.52)	92 (80)	83 (90.22)
My health in the future will depend on my medicines	297 (92.24)	104 (90.43)	106 (92.17)	87 (94.57)
My medicines protect me from becoming worse	314 (97.52)	112 (97.39)	11 (9.57)	91 (98.91)
**Concern Scale**				
Having to take medicines worries me	136 (42.24)	52 (45.22)	48 (41.74)	36 (39.13)
I sometimes worry about long-term effects of my medicines	141 (43.79)	54 (46.96)	46 (40)	41 (44.57)
My medicines are a mystery to me	79 (24.53)	33 (28.7)	28 (24.35)	18 (19.57)
My medicines disrupt my life	49 (15.22)	13 (11.3)	15 (13.04)	21 (22.83)
I Sometimes worry about becoming too dependent on my medicines	136 (42.24)	53 (46.09)	47 (40.87)	26 (28.26)
These medicines give me unpleasant side effects	135 (41.93)	51 (44.35)	50 (43.48)	34 (36.96)

## DISCUSSION

Our investigation into medication adherence among 322 patients with major non-communicable diseases in a Kathmandu-based hospital revealed that 148 (45.96%) of participants demonstrated good adherence, with 155 (48.14%) exhibiting moderate adherence according to the MMAS-4 scale. Disease-specific variations were evident, with DM patients displaying the lowest adherence at 43 (37.39%), possibly attributed to the perceived challenges of insulin injections. Conversely, COPD patients showcased the highest adherence at 49 (53.26%). The main reasons for not following medication instructions were forgetfulness, being away from home, and running out of supplies.

Importantly, the study delved into participants' beliefs about medication, with 246 (76.40%) strongly believing that medication was essential for their health and life. Approximately half ofthe participants strongly disagreed that medication disrupted their lives or had unpleasant side effects. Most participants believed that their current and future health depended on medication, reducing concerns about dependency or long-term effects, thus serving as strong motivators for adherence. Overall, the study underscores that medication adherence among non-communicable disease patients remains a challenge, with forgetfulness emerging as a primary hurdle, necessitating tailored interventions to improve adherence rates.

Disease-specific adherence variations, as seen in our study, parallel findings in Nepal by Shakya et al., where DM patients attending tertiary hospitals exhibited a higher adherence of 61%, with improved adherence linked to higher formal education and attendance at diabetes counselling.^9^ Similar study by Shrestha et al reported forgetfulness as the main cause of nonadherence in DM patients from Nepal.^10^ Similarly, Yadave et al also noted forgetfulness and feeling well as the primary causes of non-adherence in COPD patients in Nepal and counselling helped improve adherence.^11^

Pokhrel et al conducted a systematic review and metaanalysis of 14 studies involving 3276 patients from Nepaltounderstandnon-adherence toanti-hypertensive medication.^12^ They noted that non-adherence rates varied from 15.3% to 80.5%, with an overall pooled prevalence of 49% which is similar to rates in our study. The key factors linked to non-adherence in this study were forgetfulness, carelessness, medication costs, and the presence of multiple health conditions. Notably, patients in rural areas showed a higher nonadherence rate.

Our study's medication adherence rate, aligning closely with global averages, reflects the challenges highlighted by the World Health Organization's report, which reports an average adherence of 50% in chronic therapies globally.^13^

Ongoing researchers are actively looking into ways to improve medical adherence. Non-adherence can be broadly categorised as intentional and unintentional. Intentional nonadherence is when someone chooses not to take medicine on purpose, often because of feelings or beliefs about potential side effects. Unintentional nonadherence occurs when patients intend to follow their prescribed treatment but face challenges like forgetfulness or logistic issues like lack of medicine. A multifaceted approach is crucial for addressing medication adherencechallenges. Patient education and counselling sessions address intentional nonadherence, while interventions like supervised drug administration, community-based nursing, or reminder messages/apps are beneficial for unintentional nonadherence.

A Cochrane review, which included 182 randomised controlled trials on adherence, reported that methods such as telephonic follow-up, daily home visits, and family/health-worker-supervised medicine administration were effective in improving adherence. On the other hand, approaches like health education, counselling, and cognitive-behavioural therapy, while extensively studied, showed overall small and inconsistent effects in improving adherence.^14^ Novel technology-based strategy like short message services and smartphone reminder apps can become useful strategy that may be recommended especially to nonadherent patients.^15,16^

Medical doctors may face heavy workloads and time constraints, contributing to a lack of sufficient time for counselling about prescription medicine. In response, allied health workers like pharmacists and nurses can play a crucial role in medical education and drug counselling.^17^ Community health worker and pharmacist-led education-based intervention, along with regular follow-up and use of technology-based interventions like smartphone reminder apps have proven to be promising interventions to improve medication adherence in chronic diseases.^18^

The study has a few limitations worth noting. First, relying on patients to report their medication adherence may lead to inaccuracies and bias due to memory recall. Second, because the study was conducted in a hospital and used a convenience sampling method, there might be biases in the selection of participants, making it challenging to apply the findings to a broader population. Third, because the study was cross-sectional and thus lacked follow-up assessments. Additionally, the study didn't distinguish between different types of medications, making it difficult to pinpoint specific challenges related to particular classes of medications.

Despite these constraints, the study provides foundational insights into non-adherence among patients with non-communicable diseases in Nepal, laying the groundwork for future research and targeted interventions.

## CONCLUSIONS

This study found that less than half of the participants fully adhered to prescribed medications, with forgetfulness identified as the primary reason for noncompliance. Despite this, a majority expressed a strong belief in the protective benefits of medications for their current and future health.
